# Cancer stem cells are the cause of drug resistance in multiple myeloma: fact or fiction?

**DOI:** 10.18632/oncotarget.5800

**Published:** 2015-09-22

**Authors:** Reinaldo Franqui-Machin, Erik B. Wendlandt, Siegfried Janz, Fenghuang Zhan, Guido Tricot

**Affiliations:** ^1^ Interdisciplinary Graduate Program in Molecular and Cellular Biology, The University of Iowa Carver College of Medicine, Iowa City, Iowa, USA; ^2^ Department of Internal Medicine, The University of Iowa Carver College of Medicine, Iowa City, Iowa, USA; ^3^ Department of Pathology, The University of Iowa Carver College of Medicine, Iowa City, Iowa, USA

**Keywords:** drug resistance, neoplasia, myeloma and other plasma cell dyscrasias, signaling therapies

## Abstract

Multiple myeloma (MM) remains a largely incurable, genetically heterogeneous plasma-cell malignancy that contains – just like many other cancers – a small fraction of clonogenic stem cell-like cells that exhibit pronounced self-renewal and differentiation capacities, but also pronounced drug resistance. These MM stem cells (MMSCs) are a controversial but highly significant issue in myeloma research because, in our opinion, they are at the root of the failure of anti-neoplastic chemotherapies to transform myeloma to a manageable chronic disease. Several markers including CD138^−^, ALDH1^+^ and SP have been used to identify MMSCs; however, no single marker is reliable for the isolation of MMSC. Nonetheless, it is now known that MMSCs depend on self-renewal and pro-survival pathways, such as AKT, Wnt/β-catenin, Notch and Hedgehog, which can be targeted with novel drugs that have shown promise in pre-clinical and clinical trials. Here, we review the pathways of myeloma “stemness”, the interactions with the bone marrow microenvironment that promote drug resistance, and the obstacles that must be overcome to eradicate MMSCs and make myeloma a curable disease.

## INTRODUCTION

MM, the second most common blood cancer (13%), is a genetically complex, clonal plasma-cell malignancy that accounts for 1% of all newly diagnosed neoplasms [1-3]. It is characterized by uncontrolled accumulation and growth of aberrant bone marrow (BM) plasma cells, which show hallmarks of pronounced genomic instability, including DNA point mutations, deletions and amplifications across the genome, ploidy changes, and, in approximately half of cases, chromosomal translocations that rearrange the immunoglobulin heavy chain (IgH) locus [4-8]. Myelomagenesis is a multistep process that begins as asymptomatic monoclonal gammopathy of undetermined significance (MGUS), evolves to smoldering MM (SMM), continues to progress to symptomatic disease as defined by clinical and laboratory criteria, and end organ damage [9], and eventually leads to an aggressive, refractory neoplasia, comparable to blast crisis in CML or Richter's syndrome in CLL. This end stage is characterized by extramedullary tumor manifestation, elevated serum levels of LDH, complex chromosomal abnormalities on metaphase cytogenetics, and pronounced drug resistance. Two recent studies have provided strong evidence that MM is preceded by MGUS in virtually all cases [10, 11].

## MM CONTAINS CANCER STEM CELL (CSC)-LIKE CELLS

CSCs, a rare subpopulation (<1%) of tumor cells, are believed to drive drug resistance and disease relapse in many cancers [12-14]. Although the concept of CSCs has been around for decades, definitive proof for their existence was only provided in 1997 when Bonnet and Dick reported propagation of acute myeloid leukemia (AML) in immunodeficient mice [15]. The investigators showed that the tumorigenic potential of AML was restricted to a rare CD34^+^CD38^−^ cell fraction, but the tumorigenic potential was not present in ordinary “bulk” leukemia cells [15]. Subsequently, researchers identified CSCs in a host of solid and hematological tumors, including MM [12, 13,16, 17]. As early as 1981, based on studies with both myeloma cell lines and tumor samples from patients with myeloma, Drewinko *et al.* postulated that myeloma exhibits self-renewal capacity [18]. Although 35 years have passed since this seminal paper was published, many details about the exact nature and biological properties of MMSCs remain elusive.

One area of contention is the differentiation stage of the B cell in which “myeloma stemness” resides. Early work indicated that MMSCs are post-germinal center B-cells, either memory B-cells or plasmablasts [19-21]. This was based on the detection of clonotypic somatic mutations in the CDRs (complementarity determining region) of immunoglobulin heavy- and light-chain genes in myeloma cells and peripheral-blood B-cells and is compatible with evidence that MMSCs do not express the classic myeloma marker, CD138, on the cell surface, express less CD38 than conventional or “bulk” myeloma cells [22, 23], exhibit a CD19^+^CD24^+^CD27^+^ memory B-cell phenotype [24, 25], and are enriched in the B-cell fraction of the myeloma SP [26, 27]. It may be safe to assume that CDR mutations in myeloma were acquired in a T cell-dependent germinal center (GC) manner in which normal B cell precursors participated in B-cell differentiation and subsequently became neoplastic. However, it is by no means certain that this post-germinal B cell compartment indeed serves as the reservoir of MMSC. An alternative hypothesis postulates that MMSCs are part of the clonotypic plasma cell SP [28, 29] and exhibit increased levels of ALDH1A1 as well as the multidrug transporter, ABCG2/BCRP [30, 31].

## ACQUIRED DRUG RESISTANCE IS THE ROOT CAUSE OF TREATMENT FAILURE IN MYELOMA

**Figure 1:**
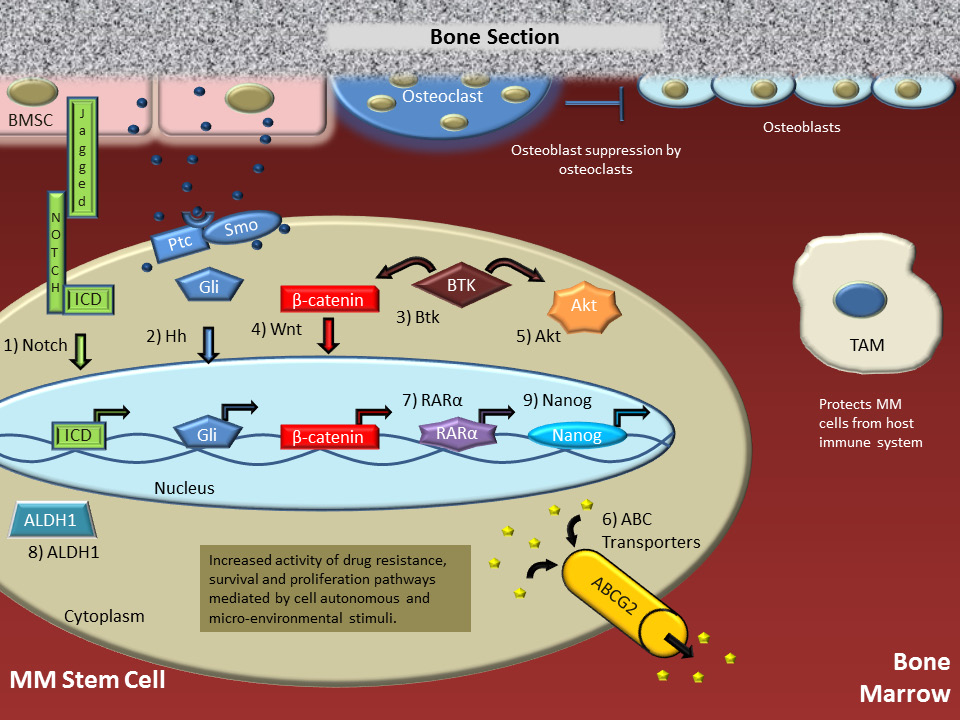
Putative multiple myeloma stem cell (MMSC) in the bone marrow microenvironment The MMSC receives both tumor cell-autonomous (autocrine) signals and a plethora of para- and juxtacrine signals from a variety of bystander cells in the tumor microenvironment (TME). Collectively, these signals govern the self-renewal and survival of the MMSC, the maintenance of its cancer stem cell (CSC) state and, importantly, the acquisition of drug resistance. This is accomplished by engaging the following signaling pathways in MMSCs: (1) Notch ligands produced by bone marrow stroma cells (BMSCs) activate a Notch receptor on the MMSC. This leads to cleavage of the receptor's intra-cellular domain (ICD) by a γ-secretase, translocation of ICD to the nucleus, and ICD-dependent activation of genes involved in self-renewal, proliferation and activation of AKT. (2) Binding of Hh signals from auto- and/or paracrine sources to Hh receptors leads to de-inhibition of Smothened (Smo), which results in activation of Gli, its translocation to the nucleus, and execution of the Gli-dependent gene expression program. (3) BTK, which is not expressed in normal plasma cells, is often aberrantly expressed in myeloma including MMSCs. Activation of BTK by targeted phosphorylation in the plasma membrane leads to cross-signaling with the WNT/b-catenin (4) and AKT (5) pathways. Our research has shown that active BTK in CSC-like myeloma cells results in increased amounts of nuclear b-catenin, pAKT, ABC transporter drug efflux ability (6) and Nanog (7). Two additional stemness factors that increase the clonogenic potential of myeloma are RARα2 (8) and ALDH1 (9). Working in concert, in ways that are poorly understood, the pathways described above increase drug resistance in myeloma and cause relapse with aggressive disease. Thus, the design and testing of MMSC-targeted therapies should be a priority in myeloma.

Numerous drugs have been evaluated in MM. These include the antineoplastic alkylating agents, cyclophosphamide, busulfan, BCNU and melphalan, the pleiotropic immunomodulator, thalidomide, and its derivatives, corticosteroids, including dexamethasone, microtubule-targeting agents, such as paclitaxel and the vinca alkaloids, and the proteasome inhibitors, bortezomib and carfilzomib [32, 33]. Although an excellent initial response to these drugs is often observed, relapse is unfortunately virtually inevitable. This experience, underlined by a large body of research demonstrating that the above-mentioned agents, with the exception of alkylators given at myeloablative doses, are ineffective in killing the most drug-resistant MM cells [34], points to MMSCs as the Achilles heel of current treatment. Therefore, MMSC-targeted therapies should be a priority in myeloma. Figure 1 depicts what we believe is a major underlying reason for acquired drug resistance in myeloma: crosstalk between canonical and non-canonical signaling pathways in myeloma cells and microenvironment that regulate the growth and survival of MMSCs. The Wingless (WNT) pathway, which is highly active in MMSCs and hematopoietic stem cells (HSC) [35], increases “stemness”. The Hedgehog (Hh) pathway, which is elevated in CD138^−^ MM cells [36], shares target genes with WNT; e.g., the D cyclins, which are important myeloma drivers [37, 38]. The Notch pathway promotes stemness, osteoclastogenesis and angiogenesis; it also activates the NFκB pathway, critical for survival, and the AKT pathway, a crucial regulator of myeloma proliferation, homing and anti-apoptotic activity, using a mechanism that includes both the silencing of the AKT inhibitor PTEN [39] and PTEN-independent changes [40]. MMSCs are further characterized by high drug-efflux capacity, a common feature of stem cells [34]. Accumulating evidence indicates that targeting the above-mentioned pathways individually in myeloma will not be effective; instead, drug cocktails inhibiting several pathways in concert will be necessary.

## THE MYELOMA SP HARBORS MULTI-DRUG RESISTANT MMSCS

Flow cytometric analysis of most if not all cancers, including MM, will readily identify a small fraction of tumor cells that is distinct from the main population by virtue of functional parameters. This is still an imperfect method, yet the most reliable one at this juncture, because it does not rely upon membrane markers, which can change depending on the microenvironment. Just like in many other types of cancer, the increased drug/dye efflux potential of MMSCs is likely to be instrumental in the acquisition of drug resistance in myeloma [41]. Loh *et al.* surveyed 21 myelomas for SP cells and found those in 18 (86%) cases with a frequency of up to 4.9% [42]. We have observed an increased frequency of these SP cells in multiple relapsed patients.

Early studies on the mechanism of drug resistance in SP cells concentrated on multidrug resistance (MDR) pumps [43], particularly MDR1, i.e., P-glycoprotein. Initial results suggested that MDR1 promotes stem cell expansion via protection from apoptosis [44], but a more recent study by *Zhou et al.* demonstrated that MDR1 is expressed in a more restricted manner than previously thought and MDR1-deficient mice contain normal numbers of SP cells [45]. These investigators attributed the SP efflux phenotype to another member of the ABC (ATP-binding cassette) transporter superfamily, ABCG2. Consistent with that, overexpression of ABCG2 in bone marrow cells resulted in an increased SP fraction. In myeloma, inhibition of ABCG2 by small molecule drugs, such as cyclosporine analogs and verapamil, proved to be ineffective, despite promising pre-clinical results suggesting that this inhibition may re-sensitize myeloma cells to vincristine, doxorubicin and dexamethasone (VAD) and, thereby, prevent tumor relapse [46]. Hirschmann-Jax *et al.* have recently implicated ABCA3, another ABC transporter, in drug efflux pathways in stem cells [31]. Although up-regulation of MDR transporter genes has been observed at relapse in patients with myeloma, additional research is warranted to elucidate drug export pathways in the course of tumor progression in myeloma and their relationship with MMSCs.

Heightened drug efflux activity is unlikely to be the sole reason for drug resistance in myeloma. In a recent study on the expression of genes encoding cancer testis antigens (CTAs), Wen *et al.* found that these genes were up-regulated in the SP fraction obtained from both human myeloma cell lines (HMCLs) and primary myeloma samples [47]. Interestingly, one CTA, LUZP7, was essential for drug resistance in myeloma cells.

## ALDH1A1 A MEDIATOR OF DRUG RESISTANCE IN MMSCS

Aldehyde dehydrogenase (ALDH) activity is key for metabolic detoxification pathways of aldehydes derived from ethanol, retinoids, cyclophosphamide and countless other substrates [48]. Among the various isomers of this family of enzymes, ALDH1 seems to play the most prominent role in CSCs [49]. ALDH1 is commonly deregulated in solid tumors and hematological malignancies [50]. It is also expressed at high levels in normal murine and human HSCs [34, 49]. Increased expression of ALDH1 has been associated with drug resistance and poor clinical outcome in cancer [51]. Our interest in ALDH1 was sparked by the possibility to use this protein as a biomarker to increase the efficiency with which myeloma cells with increased stemness (i.e., MMSCs) can be identified in and fractionated from the SP of HMCLs and primary tumor samples. However, we found very little overlap between SP and ALDH1-positive cells. Additionally, we sought to elucidate the mechanism with which ALDH1 drives drug resistance in myeloma. Our studies showed that expression of ALDH1A1, the predominant isoform of ALDH1 in myeloma cells, leads to up-regulation of 9-cis retinoic acid (9CRA) [52]. Expression of the retinoic acid receptor α (RARα) remained unchanged. Elevation of 9CRA results in increased expression of NEK2, which we found to increase both chromosomal instability and WNT/AKT-dependent drug resistance of myeloma cells [53]. In a follow-up study, we demonstrated that NEK2-induced drug resistance also resulted in the up-regulation of the drug efflux pump ABCB2 [53]. In keeping with our findings in MM, expression of ALDH1 in prostate cancer is positively correlated with stem cell markers, regulated by WNT/β-catenin signaling and associated with increased protection of CSC-like cells from chemotherapeutic attack [54].

### Retinoic Acid Receptor Alpha 2 (RARα2) a potential MMSC biomarker

Retinoic acid receptors (RARs) are a family of nuclear receptor proteins that mediate the effects of retinoic acid (RA). The RA signaling cascade, which regulates the growth and differentiation of normal and transformed cells, governs a range of biological processes including embryonic development and reproduction [55]. RA signaling occurs through one of six nuclear receptors - denoted RARα, RARβ, RARγ, RXRα, RXRβ and RXRγ. Each RAR gene encodes different transcripts due to alternative splicing. RARs are activated by two ligands, all-trans retinoic acid (ATRA) and 9CRA, and function as transcription factors [56]. RARα was the first family member tightly associated with cancer, based on findings that virtually all cases of acute promyelocytic leukemia (APL) harbor a chromosomal t(15;17) translocation that results in an oncogenic RARα-PML fusion protein which promotes cell growth and proliferation at the expense of differentiation [57]. Our research has identified RARα2 as an important marker of MMSCs [58]. Its expression is elevated in CD138^−^ cells compared to the bulk myeloma cells. Enforced expression of RARα2 induced a MMSC phenotype in myeloma cells with increased SP fraction, drug resistance, clonogenic growth, ALDH1 activity and expression of embryonic stem cell genes (e.g., Nanog, Oct 4, Sox2). Signaling pathways typically activated in CSCs (Wnt, Hh, Notch) were also elevated. Treatment of MMSCs with ATRA caused apoptotic cell death, which could be further increased by adding a Cox2 inhibitor (down-regulates Wnt signaling) and/or cyclopamine (inhibits Hh signaling). Consistent with these results, enforced lentiviral expression of RARα2 in myeloma cells that do not express the gene under normal conditions sensitized the cells to ATRA [58]. Moreover, in newly diagnosed myeloma patients, those expressing RARα2 at baseline had a significantly inferior outcome. Interestingly, these patients demonstrated the same gene expression signatures that we observed in MMSCs.

## BRUTON'S TYROSINE KINASE (BTK) IN MMSC MIGRATION AND ACTIVATOR OF WNT, AKT AND NOTCH

BTK is a non-receptor tyrosine kinase, the functional loss of which causes the hereditary immunodeficiency, X-linked agammaglobulinemia (XLA) - a disease that is characterized by a complete lack of mature B-lymphocytes and, consequently, serum immunoglobulins [59]. BTK plays a crucial role in malignancies of the mature B-lineage, in which it serves as a therapeutic target for the small-molecule inhibitor, ibrutinib. This drug has greatly improved treatment options and outcome of chronic lymphocytic leukemia (CLL), Waldenstrom macroglobulinemia (WM) and mantle cell lymphoma (MCL) [60, 61] and is now undergoing clinical testing for relapsed/refractory MM. BTK is not expressed in normal plasma cells, but is key for BCR (B-cell receptor) signaling pathways in B-lymphocytes [59, 62]. In myeloma, up-regulation of BTK leads to increased bone resorption and homing of MM cell to the BM [63].

Recent findings from our group have strongly implicated BTK in MMSC maintenance and drug resistance pathways [62, 64]. Thus, we found that BTK levels in putative MMSCs are significantly higher than in bulk myeloma cells. Drug-inducible over-expression of BTK in HMCLs with low baseline levels, led to the up-regulation of the CSC markers mentioned above, including Nanog, a master regulator of the normal and malignant stem cell program [62]. Approximately 80% of MM patients express BTK in the plasma cells [62]. In agreement with these results, a recent study by Yaccoby et. al. [65] demonstrated that silencing BTK in myeloma cells increased the growth of individual tumor nodules, yet decreased the tumor's metastatic potential suggesting that inhibition of MMSCs with the self-renewing but relatively quiescent capacity are critical for myeloma dissemination [66].

BTK may promote B-cell migration via activation of the CXCR4-CXCL12 (SDF-1) axis [67]. CXCL12 is a pleiotropic chemo-attractant for CXCR4-expressing cells, including HSCs and MMSCs, and retains these cells in the BM. Because myeloma SP cells up-regulate CXCR4, they may enjoy increased micro-environmental protection relative to bulk myeloma cells. CXCR4 blockers such as AMD3100, have been shown to release CSCs from the BM niche, thus reducing their intrinsic resistance to drugs [68]. VLA-4 is another receptor on myeloma cells that upon binding to VCAM-1 on BMSCs, promotes homing to the BM. The VLA-4 and VCAM-1 axis is also functional in HSCs and inhibiting this axis for therapeutic purposes in myeloma may cause unwelcome side effects; the same is also true for AMD3100 [69]. One study showed that lenalidomide inhibits the interaction of the myeloma SP with BMSCs [70]. However, the SP fraction in that study did not correlate with CD138^−^ status and the CD138^−^ fraction was not affected by treatment with lenalidomide. Similarly, bortezomib, a potent proteasome inhibitor has been shown to target SP cells in some studies, but was ineffective in killing CD138^−^ cells [71].

The available data support the contention that BTK is a promising therapeutic target in myeloma. Clearly, BTK is upstream of several “stemness” pathways in myeloma, including WNT, Notch and AKT, but agreement does not exist whether BTK activates or inhibits Wnt/β-catenin signaling [62, 72]. Similarly, by virtue of activating AKT, BTK may unleash a plethora of changes in myeloma cells, including increased survival via regulation of BCL-2 family proteins, elevated drug efflux activity via induction of ABCB1 and rearrangement of the cytoskeleton via inhibition of GSK [62, 73]. However, which of these changes matter to MMSCs in ways that can be therapeutically exploited, remains to be determined.

## NANOG, A TRANSCRIPTION FACTOR SWAYED BY MANY MMSC PATHWAYS

Our group has demonstrated that Nanog is a downstream target of both RARα and BTK signaling, two prominent features of MMSCs [58, 62]. Under normal physiological conditions, Nanog is a transcription factor that, along with other core transcription factors, such as Oct4 and Sox2, sustains pluripotency in embryonic stem cells. In cancer cells, NANOG drives the epithelial to mesenchymal cell transition (EMT), the expression of other “stemness” genes, and the potential of tumors to metastasize. The molecular mechanism of NANOG expression and protein activity in normal and malignant cells has not yet been elucidated. Based on our data, overexpression of BTK leads to increased levels of NANOG in myeloma cells [62]. Our most recent, unpublished work indicates that up-regulation of BTK in myeloma cells leads to phosphorylation and activation of STAT3, a crucial regulator of NANOG. Additional regulators of NANOG in myeloma are Hh and Wnt, two “stemness” pathways that are governed, at least in part, by BTK [62]. Although many details have yet to be filled in, it is possible that NANOG-dependent myeloma stemness may be therapeutically targeted using small-molecule BTK inhibitors, such as ibrutinib.

## INTERACTION OF MMSC WITH BM MICROENVIRONMENT

In MM, the microenvironment is comprised of BM stromal cells (BMSCs), osteoclasts, osteoblasts, immunity cells like macrophages, mesenchymal stem cells and others [74]. BMSCs can directly stimulate MM cell survival by direct cell-cell contact; this leads to the secretion of BMSC-derived IL-6, IGF-1, RANKL, TNF-α and other myeloma-promoting cytokines. MM cells are also known to stimulate osteoclast activation and suppress osteoblast differentiation, creating an imbalance in bone homeostasis that ultimately results in bone lesions [74]. Interestingly, when examining a stem-like MM cell population in mice, only the MM stem-like cells engrafted in the BM osteoblastic niche were capable of generating colonies after being harvested [75]. This suggests that the microenvironment has a strong influence on the MMSC population and their tumorigenic capabilities. Tumor-associated macrophages (TAM) have been described in the BM of MM patients. These modified macrophages permit the escape of tumor cells from the host immune system, a process termed immune tolerance. TAMs like endothelial progenitor cells also contribute to angiogenesis within the MM microenvironment [74]. In healthy bone marrow, other pathways like parathyroid hormone (PTH) signaling and oxygen-dependent pathways like HIF-1 sustain hematopoietic stem cells and these could potentially be hijacked by the MMSCs for their survival [76]. The tumor microenvironment and MMSCs interact in a way that protects and promotes the survival of the MMSC. This emphasizes our need to develop preclinical models that encompass all of the aspects of MM.

## TARGETING MMSCS TO OVERCOME DRUG RESISTANCE IN MYELOMA

The Hh pathway, which is highly active during embryonic and fetal development, is only sustained in those mature tissues that have a stem cell compartment [77]. Matsui et al. demonstrated Hh signaling in CD138^−^ myeloma cells but not in bulk myeloma [36]. Hh ligands are secreted by BMSC [36], but it is possible that myeloma cells also produce ligands, such as Sonic Hh [78]. Thus paracrine and autocrine cytokine loops may drive Hh signaling in myeloma. Hh signaling in myeloma leads to activation of the ABCG2 drug efflux pump [79], which may render MMSCs treated with Hh inhibitors more sensitive to doxorubicin, lenalidomide and other myeloma drugs that are substrates of drug efflux pumps [80]. Pre-clinical studies on myeloma have demonstrated that Hh inhibition may exhaust tumor-initiating cells [81]. Basal cell carcinomas and other so-called Gorlin cancers demonstrate the best response to Hh-targeted therapies because they exhibit activating mutation in key components of the Hh pathway (PTCH1); however, activating mutations have not been reported in MM [82].

Notch signaling can sustain CSCs [83] and, in breast cancer, has been shown to activate ALDH1, which promotes self-renewal of tumor cells [84]. Notch-dependent up-regulation of ALDH1 has not been shown yet in MMSCs. In myeloma, the Notch pathway drives cell proliferation, angiogenesis and survival and, via complex interaction with the tumor microenvironment, contributes to *de novo* drug resistance [85, 86]. Furthermore, inhibition of Notch in myeloma hinders osteoclast activation, thereby reducing a host of paracrine and juxtacrine signals that would otherwise sustain MMSCs [87, 88]. Potent γ-secretase inhibitors, such as MK-0752 that block the Notch pathway, are currently undergoing clinical testing [89, 90]; γ -secretases cleave the intracellular domains of stimulated Notch receptors, which are then translocated to the nucleus where they function as transcription factors [91]. Even though there is solid evidence of involvement of the Notch pathway in MM, mainly through the oncogenic Notch2 receptor, no clinical trials with Notch inhibitors have been performed to our knowledge. In pre-clinical studies on myeloma, γ -secretase inhibitors demonstrate promise and synergize with established myeloma drugs, such as bortezomib [92, 93]. Antibodies, which are more specific for one of the Notch receptors or their ligands and Notch decoys have also been developed; both strategies were shown to inhibit angiogenesis and reduce bulk tumors [90].

BTK activates two important pathways in MMSCs: Wnt/β-catenin and AKT signaling [62]. Wnt is a classic “stemness” pathway that operates mainly by stabilizing β-catenin and promoting its nuclear translocation [35, 94]. AKT is an important survival pathway that counteracts apoptotic programs in MMSCs [95]. Thus, it seems plausible that BTK inhibitors, such as ibrutinib, dampen Wnt/β-catenin and AKT-dependent mechanisms of myeloma “stemness”. An interesting recent study reported that the anti-myeloma effects of ibrutinib may be attributed to NF-κB inhibition [61]. NF-κB assists in the maintenance of CSCs in many cancers [96], yet the clinical experience with proteasome inhibitors and IMIDs raises doubt that NF-κB inhibition will be sufficient to target MMSCs efficiently.

Complicating attempts to eliminate MMSCs is the existence of inter-convertible CD138^−/+^ MM cell populations [97], because it raises the possibility that bulk myeloma cells replenish the MMSC pool after this pool has been diminished or eliminated by chemotherapy. Consequently, curing myeloma will require a concerted approach that eradicates both bulk myeloma cells and existing stem cells.

## CONCLUDING REMARKS

Drug-resistant CSC-like cells, such as MMSCs, which very likely underlie disease relapse, are a key obstacle for curing cancer. Overcoming this obstacle in myeloma is an important but difficult-to-achieve objective because our understanding of MMSC-autonomous and bone marrow microenvironment-dependent pathways of drug resistance is still limited. Knowledge gaps remain in our comprehension of the cellular signal transduction cascades that govern the maintenance of MMSCs *in vivo*. Considering that currently available tools for monitoring bulk tumor clearance after administration of chemotherapy are not suitable to assess treatment efficacy of eradicating the MMSC pool, it will be necessary to develop better methods to measure therapy responses in the stem cell compartment that can be used as surrogate markers for success. The increasing appreciation in the myeloma community of the MMSC concept and the realization that MMSCs are best eradicated in an adjuvant setting at a relatively early stage of the treatment plan, generate optimism for the future. The design and testing of new drugs that may specifically target MMSCs, such as the BTK inhibitor ibrutinib and the Wnt, Hh and Notch inhibitors, are crucial steps to accomplish this goal.
